# Life Histories and Host-Range Evaluation of Two Chrysomelid Beetles (*Zygogramma* spp.) Released against *Tithonia rotundifolia* in South Africa

**DOI:** 10.3390/insects13030267

**Published:** 2022-03-08

**Authors:** Khethani V. Mawela, David O. Simelane, Terence Olckers

**Affiliations:** 1 Agricultural Research Council-Plant Health and Protection, Private Bag X134, Queenswood, Pretoria 0121, South Africa; simelaned@arc.agric.za; 2 School of Life Sciences, University of KwaZulu-Natal, Private Bag X01, Pietermaritzburg 3209, South Africa; olckerst@ukzn.ac.za

**Keywords:** biology, host specificity, *Zygogramma signatipennis*, *Zygogramma piceicollis*, fecundity, weed biocontrol

## Abstract

**Simple Summary:**

*Tithonia rotundifolia* (Mill.) S.F. Blake (Asteraceae) is among the three *Tithonia* species from Mexico that are invasive in many countries, including South Africa. To curb the invasiveness and negative impact of *T. rotundifolia* in South Africa, two chrysomelid beetles, *Zygogramma signatipennis* (Stål) and *Zygogramma piceicollis* (Stål), from Mexico were assessed to determine their suitability for release against this invader. Biological attributes such as a short pre-oviposition period, short egg incubation period, short lifecycle and long longevity suggest that the two beetle species could successfully establish in their introduced range. Feeding, oviposition and development of both beetle species were confined within the tribe Heliantheae, but showed a very strong preference for the invasive *T. rotundifolia*. The only non-target species that supported development to adulthood was the exotic weed *Tithonia diversifolia* (Hemsl.) A. Gray, itself a target for biocontrol. Although some sunflower (*Helianthus annuus* L.) cultivars were partially utilized during host-specificity tests, none supported complete development. Since their potential threats to *H. annuus* cultivars are minimal, both *Zygogramma* species were cleared for release in South Africa in 2014.

**Abstract:**

*Tithonia rotundifolia* (Mill.) S.F. Blake (Asteraceae) is among the three *Tithonia* species from Mexico that are invasive in South Africa. To curb its invasiveness and negative impact in South Africa, two chrysomelid beetles, *Zygogramma signatipennis* (Stål) and *Zygogramma piceicollis* (Stål), from Mexico were investigated as candidate biological control agents. The life histories and host ranges of these beetles were studied under laboratory conditions to determine their suitability for release. The two beetle species displayed very similar life histories, including a short pre-oviposition period (13–14 days), incubation period (4–5 days) and lifecycle (40–45 days). The longevity of *Z. signatipennis* and *Z. piceicollis* was 113 and 125 days, while their fecundities were 1146 and 1133 eggs per female, respectively. Feeding, oviposition and development of both beetle species were confined within the tribe Heliantheae, but showed a very strong preference for the invasive *T. rotundifolia*. The only non-target species that supported development to adulthood was the exotic weed *Tithonia diversifolia* (Hemsl.) A. Gray, itself a target for biocontrol. Although some sunflower (*Helianthus annuus* L.) cultivars were partially utilized during host-specificity tests, none supported complete development, suggesting that both *Zygogramma* species are suitable for release in South Africa.

## 1. Introduction

The orange-red sunflower, *Tithonia rotundifolia* (Mill.) S.F. Blake (Asteraceae), is one of three *Tithonia* species from Mexico [1] that are naturalized throughout the humid and sub-humid tropics of many countries [2,3,4,5,6,7,8]. According to the National Environmental Management and Biodiversity Act 10 of 2004 (NEMBA) and Conservation of Agricultural Resources Act 43 of 1983 (CARA) of the South African invasive species legislation, *T. rotundifolia* is classified as a Category 1b and Category 1 weed, respectively. Invasive weeds under these categories are prohibited and their control is mandatory [8]. Since its introduction during the 1900s as an ornamental plant in South Africa [3,4], *T. rotundifolia* has escaped from gardens to become invasive in several provinces, notably Gauteng, North-West, Limpopo and Mpumalanga, with scattered populations in KwaZulu-Natal [8,9]. The distribution of *T. rotundifolia* is found in areas with an elevation ranging from 300 to 1400 m. It colonizes disturbed sunny ecosystems with a high water table, particularly open fields, disturbed abandoned sites and along railways and roads [6,8]. 

*Tithonia rotundifolia* is distinct from other *Tithonia* species, comprising an erect herbaceous annual plant that can reach over 3 m in height, with round, green to purple stems, lobed alternate leaves and orange to red sunflower-like inflorescences that are held singly at the tip [2,6]. High seed output renders *T. rotundifolia* more competitive than indigenous plants, transforming landscapes into large monospecific stands [6]. With no registered herbicides to control it, escalating invasions pose increasing threats to biodiversity and the ecological integrity of natural systems, as well as to agricultural and forestry systems in South Africa [9].

A biological control programme was thus initiated in 2007 to curb the invasiveness of *T. rotundifolia* in South Africa [9]. During 2007 and 2008, initial surveys for insect natural enemies of *T. rotundifolia* were conducted in Mexico. Amongst others, two highly damaging leaf-feeding beetles, *Zygogramma signatipennis* (Stål) and *Zygogramma piceicollis* (Stål) (Coleoptera: Chrysomelidae), were subsequently collected and imported into quarantine in South Africa for further evaluation [9]. The family Chrysomelidae is a renowned source of weed biocontrol agents [10,11] because it includes many species that are highly host specific and damaging [12]. Since chrysomelid larvae and the adults occupy the same niche, their impact on their host plants is extensive, often causing complete defoliation. The genus *Zygogramma* includes about 90 species that are known to feed mainly on plant species within the families Asteraceae and Malvaceae [13]. At least two species within the genus *Zygogramma* have been used as biocontrol agents of invasive alien weeds in India, Australia, Russia and China, with notable impact [14,15,16,17,18,19]. *Zygogramma bicolorata* Pallister caused over 90% defoliation of *Parthenium hysterophorus* L. (Asteraceae) in Australia, which resulted in a reduction of plant growth, flower production, soil seed banks and seedling emergence in the following season [17]. Similar success with *Z. bicolorata* on *P. hysterophorus* was reported in India [20]. *Zygogramma suturalis* Fabricius, which was introduced into Russia in 1979 and later into China in 1997 as a biocontrol agent of the common ragweed, *Ambrosia artemisiifolia* L. (Asteraceae), has delivered a similar impact [14,15,19].

The decision for choosing an effective biocontrol agent is influenced by many factors, including the suite of agents available and the niche targeted in order to achieve the anticipated impact on the target weed [21]. Knowledge of the life history and host range of the candidate agents is of paramount importance in the initiation of biological control programmes [22]. Besides its host specificity, the suitability of a potential biocontrol agent is determined by a combination of attributes, including its taxonomy, rate of population increase, number of generations per year, as well as its distribution and abundance in the native range [23,24,25].

In this study, the life history parameters of the two *Zygogramma* species, which included their feeding behaviour, duration of development, pre-oviposition period, fecundity and longevity, were studied to determine their positive attributes as biological control agents. Furthermore, host-specificity testing was undertaken to determine their suitability for release against *T. rotundifolia* in South Africa.

## 2. Materials and Methods

### 2.1. Cultures of Z. signatipennis and Z. piceicollis 

Cultures of *Z. signatipennis* and *Z. piceicollis* were established from individuals that were collected during surveys in Mexico. *Zygogramma signatipennis* was initially collected in Mexico City in 2007 on a closely related species, *Tithonia tubaeformis* (Jacq.) Cass. (Asteraceae), and then recorded later on *T. rotundifolia* in the Oaxaca and Chiapas provinces. *Zygogramma piceicollis* was collected for the first time in 2008 on *T. rotundifolia* in the warm and humid coast of Oaxaca Province. The two *Zygogramma* beetles were identified by Dr. Santiago Zaragoza Caballero of Universidad Nacional Autónoma de México—Instituto de Biologia (UNAM-Instituto de Biologia). The voucher specimens were deposited at the National Collection of Insects housed at the Biosystematics Division of the Agricultural Research Council-Plant Health and Protection (South Africa) and at the Instituto de Biología (Mexico). Morphologically, the two *Zygogramma* species are very similar, with slight differences. *Zygogramma signatipennis* is slightly larger in size (5.69 mm in length) and shiny black with silver green markings on the elytra. In contrast, *Z. piceicollis* is smaller (5.17 mm in length), with a dark red head and thorax and light grey markings on the elytra.

The two *Zygogramma* species were reared on *T. rotundifolia* plants that were propagated from seeds collected at several sites in Pretoria, Gauteng Province. The plants were grown in standard soil mixture composed of sand, loam, compost and vermiculite at a ratio of 1:1:1:1. Plants were irrigated with an automated overheard irrigation system and synthetic NPK fertilizer (2:3:2 (14%)) was applied once every 2 weeks before the plants were used in the experiments. When the plants reached a height of approximately 0.3 m with a canopy of about eight leaves per plant, they were exposed to newly emerged *Zygogramma* adults in gauze-covered cages (0.5 × 0.5 × 0.95 m). The gauze of the cages was made of strands that are 0.24 mm thick and with openings of 0.84 mm (diagonal), 0.24 mm (horizontal) and 0.72 mm (vertical). Newly emerged adults were confined with at least five potted *T. rotundifolia* plants in a cage. To avoid overexploitation of the leaves, the beetles were transferred to another cage with fresh plants after 10 days. As the larvae developed and the leaf damage intensified, fresh plants were added into the cages to supplement the food until all late-instar larvae had burrowed into the soil around the plants to pupate. Newly emerged adults were collected as they emerged from the soil and were used during the experiments.

### 2.2. Laboratory Conditions

All studies were conducted in a quarantine facility at the Rietondale campus of the Agricultural Research Council—Plant Health and Protection (ARC-PHP) in Pretoria. Quarantine glasshouse temperatures of 22 to 30 °C, and relative humidity of 23 to 88%, were maintained during insect rearing and throughout the laboratory studies. A photoperiod of 12–14L:12–10D was maintained and light was supplemented during winter using artificial lighting.

### 2.3. Life History Studies of Zygogramma signatipennis and Z. piceicollis

Studies on the life history of the two *Zygogramma* species included the following parameters: pre-oviposition period, egg incubation period, duration of larval development and pupation, generation time, adult longevity and fecundity. Pre-oviposition period was determined by exposing a mating pair of newly emerged adults (<24 h old) to a fresh cut leaf of *T. rotundifolia* in a transparent plastic container (80 mL). The lid of the container was removed and replaced with a gauze covering to allow ventilation. The petiole of each leaf was covered with moistened cotton wool to keep it fresh. The beetles were observed twice a day until the first eggs were deposited. Pre-oviposition period was estimated as the time between adult emergence and the onset of oviposition. The experiment was replicated at least 11 times for each *Zygogramma* species.

To determine the egg incubation period and duration of development of the immature stages to adulthood, leaves of *T. rotundifolia* containing at least 20 newly laid (<24 h old) eggs were collected from the main culture of each beetle species and placed in a ventilated plastic container as described above. Egg incubation period was determined as the time taken for 20 eggs to hatch. Each hatching larva was transferred to a fresh-cut leaf of *T. rotundifolia* and confined singly in a well-ventilated plastic container. The development of each larva was monitored, with observations done twice a day until the adult stage was reached. At the late larval stage, moistened vermiculite was added into each container to facilitate pupation. The duration of larval development was determined as the time between hatching and pupation, with the time between pupation and adult emergence similarly recorded. The total generation time was estimated as the number of days between P_1_ adult emergence and the emergence of their F_1_ adult progeny,

To determine adult longevity and fecundity, each pair of newly emerged (<24 h old) adults of each *Zygogramma* species (1 male:1 female) was confined with cut leaves of *T. rotundifolia* placed in a ventilated plastic container. The petioles were wrapped with moistened cotton wool to keep the leaves fresh. The pairs were allowed to mate and deposit eggs on the leaves. After every 24 h, the eggs were counted, and the leaf replaced daily until both male and female beetles had died. Longevity was determined as the average number of days that the male and female beetles survived, while fecundity was determined as the total number of eggs deposited per female during its lifetime. The experiment was replicated 12 times for each *Zygogramma* species.

### 2.4. Host Range of Zygogramma signatipennis and Z. piceicollis

The host ranges of the two *Zygogramma* species were evaluated using three series of tests namely: no-choice, paired-choice and multi-choice tests. The parameters measured during these tests included adult feeding damage, oviposition, larval feeding damage and survival to adulthood. 

#### 2.4.1. Test-Plant Species

Test-plant species were grown from seedlings and/or cuttings collected from different localities in Gauteng, KwaZulu-Natal, Mpumalanga and Limpopo provinces in South Africa. Seeds of different cultivars of commercially grown sunflower were provided by Agricol (Pretoria) and the ARC-Grain Crops Institute (Potchefstroom), while ornamental plant species were purchased from different nurseries in Pretoria. 

The selection of test plants for the no-choice tests was based on their taxonomic relatedness to the genus *Tithonia* [26] (Table 1). Test plants were thus predominantly in the family Asteraceae, with a strong bias towards those in the subfamily Asteroideae, particularly in the tribe Heliantheae in which the genus *Tithonia* belongs. Other than the Heliantheae, of which seven genera are native to South Africa [27], test plants also included species in eight other tribes (Senecioneae, Calenduleae, Anthemideae, Astereae, Coreopsideae, Tageteae, Eupatorieae and Heliantheae) in the Asteroideae. Indigenous, ornamental and crop plants of economic value within and outside the family Asteraceae were also included in the list of test plants.

#### 2.4.2. No-Choice Feeding, Oviposition and Larval Development Tests

No-choice tests on the two *Zygogramma* species were conducted with 47 test-plant species in nine families to determine their suitability for feeding, oviposition and larval development. Test plants that were grown in 2.5-litre pots were washed with a jet of water to remove unwanted pests, and transferred to the quarantine glasshouse, where they were placed in separate gauze-covered cages (0.55 × 0.55 × 0.95 m). Each caged test plant was inoculated with four pairs (4 males and 4 females) of newly emerged adult beetles obtained from the main culture. After 21 days, the beetles were removed from the cage and the palatability of each test plant was evaluated by rating the feeding damage on the leaves as follows: 0 = no feeding; 1= exploratory feeding; 2 = restrained feeding (small feeding holes); and 3 = normal feeding (large feeding holes). On test-plant species where oviposition occurred, the development of larvae was monitored until the F_1_ adult progeny emerged. For each *Zygogramma* species and test-plant species, the experiment was replicated at least three times.

#### 2.4.3. Paired-Choice Feeding and Oviposition Tests

Paired-choice tests were conducted on both *Zygogramma* species to determine their preference for the natural host (*T. rotundifolia*) in the presence of a phylogenetically related test-plant species, particularly those that were utilized during no-choice tests. Two potted plants (*T. rotundifolia* and a test plant) were confined with four mating pairs of newly emerged *Zygogramma* adults in a gauze-covered cage (0.55 × 0.55 × 0.95 m). After a 21-day exposure to the plants, the beetles were removed, and the leaves of each plant species were examined to assess the degree of feeding damage and oviposition by the beetles. Feeding damage was rated as described in the no-choice tests. The paired-choice tests were replicated five times.

#### 2.4.4. Multi-Choice Feeding and Oviposition Tests

The multi-choice tests were carried out to verify the feeding and oviposition preferences of the two *Zygogramma* species when presented with closely related plant species. These tests were conducted in a large nylon-screened walk-in cage (4 × 4 × 2 m) and were intended to create conditions that were as near natural as possible. Three plants of each of the 17 test-plant species were arranged randomly in the cage, and 30 mating pairs of newly emerged *Zygogramma* adults were released into the cage. After 21 days, the beetles were removed, and the leaves of each plant were examined to determine the degree of feeding damage and number of eggs laid. The experiment was repeated a further three times, using two *Tithonia* species and four sunflower cultivars that had at least been fed on during the first round of the experiment. 

### 2.5. Statistical Analysis

Data from the biology studies and host-specificity tests were analysed with Statistica (Statistics version 13). Comparisons of the means of the life history parameters were made between the two *Zygogramma* species using Student’s *t*-test. The data from the no-choice and multi-choice tests (oviposition and adult emergence) were checked for homogeneity of variances using Levene’s test. As this test indicated equal variances, the means were compared using a one-way analysis of variance (ANOVA). Fisher’s Least Significant Difference was used to separate the means at 95% confidence level. Kruskal–Wallis tests were used to analyse the data on feeding damage scores from the no-choice and multi-choice tests. For the paired-choice tests, comparisons of the means of eggs laid and numbers of adults emerged were made between the test and control plants using Student’s *t*-test while the feeding damage scores were compared using Mann–Whitney U-tests.

## 3. Results

### 3.1. Life Histories of Zygogramma signatipennis and Z. piceicollis

The life histories of *Z. signatipennis* and *Z. piceicollis* are very similar, with the adults and larvae of both beetle species causing similar feeding patterns on leaves. The females of both *Zygogramma* species lay eggs singly under the leaf surfaces and flower buds, but can also oviposit on the stems and inflorescences when leaves have been depleted. However, the most preferred oviposition sites are shoots consisting of young leaves and/or flower buds.

The pre-oviposition period of the two *Zygogramma* species was similar and not significantly different (*t* = 1.170, df = 22, *p* = 0.254), with females taking 13 to 14 days to commence oviposition (Table 2). The mean (±SE) egg incubation period of *Z. signatipennis* (5.24 ± 0.12 days; *n* = 25) was significantly higher (*t* = 2.498, df = 45, *p* = 0.016) than that of *Z. piceicollis* (4.77 ± 0.13 days; *n* = 22). On hatching, the neonate larvae start feeding immediately, making tiny holes on the leaves and are mostly found between the axillary buds and the leaf petioles, shoot tips and flower buds. As the larvae develop, the size of the feeding holes on the leaf blades increases. 

The duration of the larval development of *Z. signatipennis* was 21.41 ± 1.09 (*n* = 17) days, which was significantly longer than the 18.13 ± 0.79 (*n* = 15) days for *Z. piceicollis* (*t* = 2.368, df = 30, *p* = 0.024) (Table 2). The late-instar larvae drop onto the ground where they burrow into the soil to pupate. The duration of the pupal stage of *Z. signatipennis* was 11.11 ± 1.09 (*n* =17) days, which was slightly longer than the 10.20 ± 0.71 (*n* = 15) days of *Z. piceicollis*, with the difference bordering on significance (*t* = 0.681, df =30, *p* = 0.05). The duration of development from egg to adult was 32.53 ± 0.75 (*n* = 17) days in *Z. signatipennis*, which was significantly longer (*t* = 3.490, df = 30, *p* = 0.002) than the 28.33 ± 0.96 (*n* = 15) days for *Z. piceicollis*. The total generation time (i.e., number of days between P_1_ adult emergence and the emergence the F_1_ adult progeny) was 45 and 41 days for *Z. signatipennis* and *Z. piceicollis*, respectively.

Adults of *Z. signatipennis* and *Z. piceicollis* lived for 112.50 ± 9.93 (*n* = 24) and 124.92 ± 13.02 (*n* = 26) days, respectively, and the differences in longevity were not significant (*t* = 0.749, df = 48, *p* = 0.457) (Table 2). There were no differences in the longevity of males and females for either beetle species. The mean fecundity was 1146.09 ± 224.99 eggs per female (*n* = 11) for *Z. signatipennis* and 1133.09 ± 146.57 eggs per female (*n* =11) for *Z. piceicollis*, with no significant difference between the two species (*t* = 0.048, df =20, *p* = 0.962). Both *Z. signatipennis* and *Z. piceicollis* females laid up to 100 eggs/day before the age of 22 days; thereafter, daily oviposition declined gradually.

### 3.2. Host Range of Zygogramma signatipennis and Z. piceicollis

#### 3.2.1. No-Choice Tests

During no-choice tests, *Z. signatipennis* and *Z. piceicollis* were subjected to a total of 47 plant species in 10 plant families (Table 1). Feeding and oviposition by both beetle species was mainly confined to species within the tribe Heliantheae of the Asteraceae (Table 3 and Table 4), with significant differences between the susceptible species.

Feeding damage by *Z. signatipennis* was rated as normal on the target weed (*T. rotundifolia*), restrained on *T. diversifolia* and exploratory on seven other plant species (*A. caffrum, H. annuus* cv. Agsun5382, *Coreopsis* sp., *Mikania natalensis, M. capensis, Conyza* sp. and *Senecio tamoides*), resulting in significant differences overall (H = 22.202; *p* = 0.022). *Zygogramma signatipennis* laid significantly more eggs on *T. rotundifolia* (*F*_5, 13_ = 7.257, *p* = 0.002), with an average of 79.67 ± 16.41 (*n* = 3) eggs versus an average of 33.25 ± 15.40 (*n* = 4) eggs on *T. diversifolia* and 16.33 ± 11.29 (*n* =3) eggs on *H. annuus* cv. Sunstripe (Table 3). Although Z. *signatipennis* deposited a few eggs on *Dahlia* sp. (Asteraceae), *Adenostemma caffrum* (Asteraceae) and *Amaranthus* sp. (Amaranthaceae), no larval development was observed on these species. *Zygogramma signatipennis* developed successfully on only the two *Tithonia* species, with an average of 42.33 ± 12.73 (*n* =3) adult progeny emerging from *T. rotundifolia* versus 5.25 ± 4.59 (*n* = 4) from *T. diversifolia* and none from *H. annuus* cv. Sunstripe. The differences in progeny production between the test-plant species were significant (*F*_5, 13_ = 9.195, *p* < 0.001).

Very similar results were obtained with *Z. piceicollis*, with normal feeding damage on the target weed (*T. rotundifolia*) and the invasive *T. diversifolia.* The beetle exhibited exploratory feeding damage on four sunflower cultivars and five other test plants (*Rudbeckia fulgida, Coreopsis* sp., *Aster novi-belgii, A. caffrum* and *Delairea odorata*) within the Asteraceae family, with scores ranging from 0.06 to 0.87 and significant overall differences between the test-plant species (H = 21.589; *p* = 0.027). Significantly more eggs were deposited (*F*_4, 10_ = 9.821, *p* = 0.002) on *T. rotundifolia* (56.33 ± 4.04; *n* = 3) than on *T. diversifolia* (29.33 ± 5.78; *n* = 3) and *H. annuus* cultivars Agsun 5551 (13.0 ± 7.81; *n* = 3), Agsun 5382 (11.0 ± 4.04; *n* = 3) and Nojana KL (1.33 ± 1.33; *n* =3) (Table 4). However, all larvae of *Z. piceicollis* died prematurely on the three sunflower cultivars and successful development was only recorded on the two weedy *Tithonia* species, with a mean of 29 and 10 adult progeny emerging from *T. rotundifolia* and *T. diversifolia*, respectively. As before, these overall differences were significant (*F*_4, 10_ = 23.880, *p* < 0.0001).

#### 3.2.2. Paired-Choice Tests

Paired-choice tests were carried out using the sunflower cultivar on which each beetle species performed best during the no-choice tests; i.e., Sunstripe for *Z. signatipennis* and Agsun 5551 for *Z. piceicollis*. *Zygogramma signatipennis* deposited 143.80 ± 9.32 (*n* = 5) eggs on *T. rotundifolia*, which was almost 20 times higher than that on *H. annuus* cv. Sunstripe (7.20 ± 1.77; *n* =5) and hence significantly different (*t* =16.067; df = 8, *p <* 0.005) (Table 5). Similar to other tests, about 60% of eggs developed to adulthood on *T. rotundifolia* versus 0% on *H. annuus* (*t* = 8.808; df = 8, *p <* 0.005). In contrast, *Z. piceicollis* avoided sunflower for oviposition (Table 5) and deposited an average of 35.43 ± 3.61 (*n* = 5) eggs on *T. rotundifolia* (*t* = 9.79561; df = 8; *p <* 0.005). Consequently, no adults of *Z. piceicollis* were reared on *H. annuus* cv. Agsun 5551, compared to 17.60 ± 1.94 beetles on *T. rotundifolia* (*t* = 9.07651; df = 8; *p <* 0.005). Both *Zygogramma* species exhibited only exploratory feeding damage on *H. annuus* but fed normally on *T. rotundifolia*, with significant differences between the test plants for both Z. *signatipennis* (U = 2.795, *p* = 0.0051) and *Z. piceicollis* (U = 2.738, *p* = 0.0061) (Mann–Whitney U-test).

#### 3.2.3. Multi-Choice Tests 

During multi-choice tests, both *Zygogramma* species consistently preferred the target weed (*T. rotundifolia*) for oviposition and feeding. *Zygogramma signatipennis* deposited over nine times more eggs on *T. rotundifolia* (74.77 ± 17.11; *n* = 9) than on *T. diversifolia* (8.00 ± 2.77.13; *n* = 9) and none on any of the sunflower cultivars or other test-plant species, with significant differences between test-plant species (*F*_4, 40_ = 17.83; *p* < 0.00001) (Table 6). Similarly, *Z. piceicollis* deposited a mean of 25.33 ± 3.68 (*n* =9) eggs on *T. rotundifolia* and none on *T. diversifolia* or any of the other test-plant species, thus displaying significant differences between test-plant species (*F*_4, 40_ =47.447, *p* < 0.00001). There were significant differences in feeding damage between test-plant species for both *Z. signatipennis* (H = 38.932; *p <* 0.00001) and *Z. piceicollis* (H = 39.001; *p <* 0.00001). Feeding damage caused by *Z. signatipennis* was rated as normal on *T. rotundifolia* and only exploratory on *T. diversifolia* and two *H. annuus* cultivars (Agsun 8251 and Nojana KL). Similarly, feeding damage caused by *Z. piceicollis* was normal on *T. rotundifolia* and only exploratory on *T. diversifolia* and two *H. annuus* cultivars (Nojana KL and Agsun5382) (Table 6). None of the 13 remaining test-plant species were fed on by either of the *Zygogramma* species (Table 6).

## 4. Discussion

Our results indicated that both *Zygogramma* species were safe for release as biological control agents for *T. rotundifolia*. The life histories of the two *Zygogramma* beetle species were somewhat similar, both exhibiting short life cycles, high female fecundity and long-lived adults, all of which are positive attributes of biocontrol agents [23,24,25]. The short life cycle and high female fecundity could enable both beetle species to sustain high populations and possibly withstand parasitism and predation in the field [28].

The pre-oviposition periods of *Z. signatipennis* and *Z. piceicollis* were longer than that of *Z. bicolorata* [29], an effective biocontrol agent [17]. However, the egg incubation period of both *Zygogramma* species was similar to that of *Z. bicolorata* [29]. Piper [30] reported that *Z. suturalis*, another effective agent, lived for about two months in the laboratory and also that females lived longer than males. The longevity of both *Z. signatipennis* and *Z. piceicollis* was 112 and 124 days in the laboratory, respectively, which was considerably longer than that of *Z. suturalis* [30] and the 80 to 85 days reported for *Z. bicolorata* [29]. In contrast to *Z. suturalis* [30], the longevity of males and females of the two *Zygogramma* species and those of *Z. bicolorata* [29] are similar. The average fecundity of *Z. signatipennis* and *Z. piceicollis* females (1133 to 1146 eggs) was also higher than that of *Z. bicolorata* (1019 eggs) [29], but twice as high as that of *Z. suturalis* (563 eggs) [30]. Both *Z. signatipennis* and *Z. piceicollis* thus have desirable biological attributes that may facilitate successful establishment in the field, similar to that displayed by *Z suturalis* [14,15] and *Z. bicolorata* [16,18,19] in their introduced ranges. 

Through host-specificity testing, the risk of releasing agents that may display non-target effects on plants of economic importance, or have a negative impact on the native flora, particularly rare and threatened species, is largely eliminated [31]. No-choice tests conducted under laboratory conditions are typically conservative as they circumvent natural host selection by insects, often resulting in the utilization of hosts that would otherwise be avoided under outdoor, free-choice conditions [32]. The range of plant species utilized under these conditions constitutes an insect’s physiological host range (i.e., the range of plant species that satisfy its feeding requirements) and thus an overestimation of its true host range [33,34]. This is contrasted with its ecological (true) host range, which constitutes the range of plant species naturally utilized, while coping with the biotic and abiotic stresses of field conditions [33,34]. Despite the unnatural caged conditions, both *Zygogramma* species displayed a high degree of host specificity, by feeding, ovipositing and developing best on the target weed. Therefore, the minimal feeding and reduced oviposition on some sunflower cultivars by both *Zygogramma* species during the no-choice tests could be regarded as laboratory artefacts that are highly unlikely to happen under natural field conditions. 

Indeed, the results of the multi-choice tests, conducted in larger walk-in cages where the beetles were able to exhibit their feeding and oviposition choices among a wide range of plant species, provided a better indication of choice under field conditions. Both beetle species displayed strong oviposition preferences for *T. rotundifolia*, with *Z. signatipennis* and *Z. piceicollis* laying 90% and 100% of their eggs, respectively, on *T. rotundifolia*. The failure of the larvae of both *Zygogramma* species to complete their development on any of the sunflower cultivars is also a clear indication that the crop is at minimal risk in South Africa. Similarly, during laboratory no-choice tests in other countries, *Z. bicolorata* and *Z. suturalis* also accepted cultivated sunflower for feeding and/or oviposition [35,36,37]. However, both *Z. suturalis* and *Z. bicolorata* were released in non-native countries and achieved considerable impact on their respective target weeds, without posing any threats to either cultivated sunflower or any non-target species [14,16,17,19]. 

Despite some concerns about possible negative effects on non-target organisms [38], classical biological control remains the most sustainable, cost-effective, environmentally friendly and internationally acceptable method of managing invasive alien species that flourish in the absence of their natural enemies in their new range [39]. This study has shown that the two *Zygogramma* species are safe for release as biocontrol agents of *T. rotundifolia* in South Africa and pose no threat to plant species that are indigenous and of economic value in this country. Permission for their release in South Africa was obtained in 2014 and both *Zygogramma* species were released during the same year. 

## Figures and Tables

**Table 1:** List of test-plant species that were used during host-specificity tests on *Zygogramma signatipennis* and *Zygogramma piceicollis*.

FAMILY Tribe	Plant Species	Status ^a^
ASTERACEAE		
Heliantheae	*Tithonia rotundifolia* (Mill.) S.F. Blake	A, I
Heliantheae	*Tithonia diversifolia* (Hemsl) A. Gray	A, I
Heliantheae	*Helianthus annuus* L. cv. Agsun 5551	A, C
Heliantheae	*Helianthus annuus* L. cv. Agsun 8251	A, C
Heliantheae	*Helianthus annuus* L. cv. Agsun 5382	A, C
Heliantheae	*Helianthus annuus* L. cv. Nojana Kl	A, C
Heliantheae	*Helianthus annuus* L. cv. Sunstripe	A, C
Heliantheae	*Helianthus tuberosus* L.	A, C
Heliantheae	*Xanthium strumarium* L.	A, I
Heliantheae	*Aspilia africana* (Pers.) C.D. Adams	N
Heliantheae	*Rudbeckia fulgida* (S.F. Blake)	A, O
Heliantheae	*Blainvillea gayana* Cass.	N
Eupatorieae	*Ageratina adenophora* (Spreng.) R.M. King & H. Rob.	A, I
Eupatorieae	*Ageratina riparia* (Regel) R.M. King &. H. Rob.	A, I
Eupatorieae	*Adenostemma caffrum* J.R. Forst. & G. Forst.	N
Eupatorieae	*Ageratum conyzoides* L.	A, I
Eupatorieae	*Mikania natalensis* DC.	N
Eupatorieae	*Mikania capensis* DC.	N
Tageteae	*Flaveria bidentis* (L.) Kuntze.	A, O
Tageteae	*Tagetes erecta* L.	A, O
Coreopsideae	*Dahlia* sp. Cav.	A, O
Coreopsideae	*Coreopsis* sp. L.	A, O
Coreopsideae	*Bidens pilosa* L.	A, I
Coreopsideae	*Bidens bipinata* L.	A, I
Anthemideae	*Artemisia afra* Jacq. ex Willd.	N
Anthemideae	*Schistostephium heptalobum* (DC.) Benth. & Hook.f.	N
Astereae	*Felicia amelloides* (L.) Voss	N
Astereae	*Aster novi-belgii* L.	A, O
Astereae	*Conyza* sp. Less.	A, I
Calenduleae	*Chrysanthemoides monilifera* (L.)	N
Calenduleae	*Dimorphotheca sinuata* DC.	N
Senecioneae	*Delairea odorata* Lem.	N
Senecioneae	*Senecio macroglossus* DC.	N
Senecioneae	*Senecio angulatus* L.f.	N
Senecioneae	*Senecio tamoides* DC.	N
Senecioneae	*Senecio barbertonicus* Klatt	N
Senecioneae	*Euryops pectinatus* (L.) Cass.	N
Arctotideae	*Arctotis arctotoides* (L.f.) O. Hoffm	N
Arctotideae	*Gazania* sp. (L.) Gaertn.	N
Cichorieae	*Lactuca sativa* L.	A, C
APIACEAE		
	*Daucas carota* L.	A, C
AMARANTHACEAE		
	*Amaranthus* sp. L.	A, I
BRASSICACEAE		
	*Brassica oleracea* var. *capitata* L.	A, C
EUPHORBIACEAE		
	*Ricinus communis* L.	A, I
FABACEAE		
	*Phaseolus vulgaris* L.	A, C
SOLANACEAE		
	*Capsicum annuum* L.	A, C
	*Solanum esculentum* L.	A, C
	*Solanum melongena* L.	A, C
	*Solanum tuberosum* L.	A, C
CHENOPODIACEAE		
	*Beta vulgaris* L.	A, C
	*Beta vulgaris* var. cicla L.	A, C
POACEAE		
	*Zea mays* L.	A, C

^a^ Status in South Africa: A = alien; C = crop; I = invasive; N = native; O = ornamental.

**Table 2:** Means ± SE (n) ^1^ of the different life history parameters of *Zygogramma signatipennis* and *Z. piceicollis* reared on *Tithonia rotundifolia*.

Species	Egg Incubation (Days)	Duration of Larva (Days)	Duration of Pupa (Days)	Duration of Egg to Adult (Days)	Pre-Oviposition (Days)	Adult Longevity (Days)	Fecundity (Eggs/Female)
*Z. signatipennis*	5.24 ± 0.13 (22) ^a^	21.41 ± 1.09 (17) ^a^	11.12 ± 1.10 (17) ^a^	32.53 ± 0.75 (17) ^a^	14.82 ± 0.96 (13) ^a^	112.50 ± 9.93 (24) ^a^	1146.09 ± 224.99 (11) ^a^
*Z. piceicollis*	4.77 ± 0.14 (25) ^b^	18.13 ± 0.79 (15) ^b^	10.20 ± 0.71 (15) ^a^	28.33 ± 0.96 (15) ^b^	13.31 ± 0.86 (11) ^a^	124.92 ± 13.02 (26) ^a^	1133.09 ± 146.57 (11) ^a^

^1^ Means within the same column followed by the same letters did not differ significantly (*p >* 0.05).

**Table 3:** Mean ± SE (n) feeding damage rating, oviposition and number of adult progeny produced by *Zygogramma signatipennis* adults on test-plant species that supported feeding and oviposition during the no-choice tests.

Plant Species	Feeding Score ^1,2^	Eggs/Plant ^2^	Adults/Plant ^2^
*Tithonia rotundifolia ^#^*	2.97 ± 0.03 (3) ^a^	81.33 ± 17.64 (3) ^a^	54.50 ± 6.50 (3) ^a^
*T. diversifolia*	1.88 ± 0.9 (4) ^b^	33.25 ± 15.40 (4) ^b^	5.25 ± 4.59 (4) ^b^
*Helianthus annuus* (Agsun 8251)	0.73 ± 0.37 (3) ^c^	0	0
*H. annuus* (Agsun 5382)	0.20 ± 0.10 (3) ^d^	0	0
*H. annuus* (Sunstripe)	0.23 ± 0.12 (3) ^d^	16.33 ± 11.29 (3) ^b^	0
*Adenostemma caffrum*	0.50 ± 0.25 (3) ^c^	1.0 ± 1.0 (3) ^c^	0
*Mikania natalensis*	0.13 ± 0.08 (3) ^d^	0	0
*M. capensis*	0.10 ± 0.06 (3) ^d^	0	0
*Dahlia* sp.	0	1.0 ± 1.0 (3) ^c^	0
*Coreopsis* sp.	0.25 ± 0.15 (4) ^d^	0	0
*Conyza* sp.	0.06 ± 0.03 (3) ^d^	0	0
*Senecio tamoides*	0.05 ± 0.02 (3) ^d^	0	0
*Amaranthus* sp.	0	0.33 ± 0.330 (3) ^c^	0

^1^ Feeding damage scores from 0 to 3: 0 = no feeding; 1= small feeding punctures (exploratory feeding); 2 = small feeding holes (restrained feeding); and 3 = large feeding holes (normal feeding). ^2^ Means followed by the same letters within columns did not differ significantly (*p* > 0.5). Zero scores were excluded from statistical analysis. ^#^ Control/target weed species.

**Table 4:** Mean ± SE (n) feeding damage rating, oviposition and number of adult progeny produced by *Zygogramma piceicollis* adults on test-plant species that supported feeding and oviposition during the no-choice tests.

Plant Species	Feeding Score ^1,2^	Eggs/Plant ^2^	Adults/Plant ^2^
*Tithonia rotundifolia ^#^*	3.0 (3) ^a^	56.33 ± 4.04 (3) ^a^	29.00 ± 4.62 (3) ^a^
*T. diversifolia*	2.50 ± 0.06 (3) ^a^	29.33 ± 5.78 (3) ^b^	10.00 ± 3.46 (3) ^b^
*Helianthus annuus* (Agsun 5551)	0.53 ± 0.27 (3) ^b^	13.01 ± 7.81 (3) ^bc^	0
*H. annuus* (Agsun 8251)	0.13 (3) ^c^	0	0
*H. annuus* (Agsun 5382)	0.33 ± 0.13 (3) ^c^	11.00 ± 4.04 (3) ^bc^	0
*H. annuus* (Nojana Kl)	0.87 ± 0.19 (3) ^b^	1.33 ± 1.33 (3) ^c^	0
*H. annuus* (Sunstripe)	0.23 ± 0.12 (3) ^c^	0	0
*Rudbeckia fulgida*	0.36 ± 0.36 (3) ^bc^	0	0
*Adenostemma caffrum*	0.33 ± 0.20 (3) ^c^	0	0
*Coreopsis* sp.	0.50 ± 0.25 (3) ^bc^	0	0
*Aster novi-belgii*	0.16 ± 0.09 (3) ^c^	0	0
*Delairea odorata*	0.06 ± 0.06 (3) ^c^	0	0

^1^ Feeding damage scores from 0 to 3: 0 = no feeding; 1= small feeding punctures (exploratory feeding); 2 = small feeding holes (restrained feeding); and 3 = large feeding holes (normal feeding). ^2^ Means followed by the same letters within columns did not differ significantly (*p >* 0.05). Zero scores were excluded from statistical analysis. ^#^ Control/target weed species.

**Table 5:** Mean ± SE (n) feeding damage, oviposition and production of adult progeny by *Zygogramma signatipennis* and *Z. piceicollis* on *Tithonia rotundifolia* and *Helianthus annuus* during the paired-choice tests.

Parameter	*Zygogramma signatipennis*		*Zygogramma piceicollis*	
	*T. rotundifolia*	*H. annuus* (Sunstripe)	*T. rotundifolia*	*H. annuus* (Agsun 5551)
Feeding damage ^1,2^	3.00 (5) ^a^	0.36 ± 0.02 (5) ^b^	2.98 ± 0.02 (5) ^a^	0.24 ± 0.02 (5) ^b^
Eggs/plant ^2^	143.80 ± 8.32 (5) ^a^	7.20 ± 1.77 (5) ^b^	35.43 ± 3.61 (5)	0
Adult progeny/plant ^2^	86.20 ± 9.79 (5)	0	17.60 ± 1.94 (5)	0

^1^ Feeding damage scores from 0 to 3: 0 = no feeding; 1= small feeding punctures (exploratory feeding); 2 = small feeding holes (restrained feeding); and 3 = large feeding holes (normal feeding). ^2^ Means followed by the same letters within rows of each beetle species did not differ significantly (*p >* 0.05). Zero scores were excluded from statistical analysis.

**Table 6:** Mean ± SE (n) oviposition and feeding damage of *Zygogramma signatipennis* and *Z. piceicollis* on various plant species during the multi-choice tests.

	*Zygogramma signatipennis*		*Zygogramma piceicollis*	
Plant Species	Eggs/Plant ^1^	Feeding Damage ^1,2^	Eggs/Plant ^1^	Feeding Damage ^1,2^
ASTERACEAE				
*T. rotundifolia* ^#^	74.77 ± 17.11 (9) ^a^	2.90 ± 0.04 (9) ^a^	25.33 ± 3.68 (9) ^a^	2.85 ± 0.05 (9) ^a^
*T. diversifolia*	8.00 ± 2.77 (9) ^b^	1.33 ± 0.12 (9) ^b^	0	1.61 ± 0.08 (9) ^b^
*H. annuus* (Sunstripe)	0	0	0	0
*H. annuus* (Nojana KL)	0	0.05 ± 0.02 (9) ^c^	0	0.50 ± 0.13 (9) ^c^
*H. annuus* (Agsun8251)	0	0.10 ± 0.06 (9) ^c^	0	0
*H. annuus* (Agsun5382)	0	0	0	0.1 ± 0.06 (9) ^d^
*A. adenophora*	0	0	0	0
*Ageratum* sp.	0	0	0	0
*Chrysanthemum* sp.	0	0	0	0
*Coreopsis* sp.	0	0	0	0
*Conyza* sp.	0	0	0	0
*R. fulgida*	0	0	0	0
*Gazania* sp.	0	0	0	0
*S. angulatus*	0	0	0	0
*L. sativa*	0	0	0	0
FABACEAE				
*P. vulgaris*	0	0	0	0
POACEAE				
*Z. mays*	0	0	0	0

^1^ Means followed by the same letters within columns did not differ significantly (*p* > 0.05). Zero scores were excluded from statistical analysis. ^2^ Feeding damage scores from 0 to 3: 0 = no feeding; 1 = small feeding punctures (exploratory feeding); 2 = small feeding holes (restrained feeding); and 3 = large feeding holes (normal feeding). ^#^ Control/target weed species.

## Data Availability

All raw data generated or analysed during this study can be obtained by contacting the corresponding author.
